# Perceptual Organization Based Upon Spatial Relationships in Alzheimer’s Disease

**DOI:** 10.1155/2003/856309

**Published:** 2003-01-01

**Authors:** Daniel D. Kurylo, Walter C. Allan, T. Edward Collins, Joshua Baron

**Affiliations:** ^1^Department of PsychologyBowdoin CollegeBrunswickMEUSA; ^2^Foundation for Blood ResearchScarboroughMEUSA; ^3^Maine Medical CenterPortlandMEUSA

## Abstract

Alzheimer’s disease (AD) is often accompanied by impaired object recognition, thereby reducing the ability to recognize common objects and familiar faces. Impaired recognition may stem from decreased efficacy in integrating visual information. Studies of perceptual abnormalities in AD indicate an impairment in organizing elements of the visual scene, thereby confusing components of individual forms. This type of impairment is consistent with the characteristics of neural loss, which impact cortical integration. To examine the extent to which perceptual organization is impaired in AD, psychophysical measurements were made of visual perceptual grouping based upon spatial relationships in a group of AD patients and demographically matched elderly control subjects. A comparison was also made between young and elderly control subjects to evaluate the effects of aging on these capacities. Deficits in perceptual organization were found for a subgroup of AD patients, which corresponded to impairment on facial recognition. A less profound functional decline was found for the elderly control group. The degree of impairment for AD subjects did not correlate to level of dementia, but instead appears to be idiosyncratic to individual patients. These results are consistent with impaired integrative function in AD, the degree of which reflects individual differences in the regional distribution of neuropathological changes.

## 1. Introduction

Numerous visual abnormalities are reported to accompany Alzheimer's disease (AD) (for review [3,8,18, 20,32,33]). Visual symptoms include impaired facial recognition [7,44], impaired discrimination or recognition of familiar objects [6,12,19,22,44], and impaired visuospatial abilities [31]. Unlike memory impairment, which is characteristic in all cases of AD, visual impairments vary in prevalence and severity. Cases range from individuals who are free from any observable visual dysfunction, to those who manifest significant impairment of object recognition or visuospatial abilities. The heterogeneity of visual function likely reflects individual differences in the regional distribution of neuropathological changes, an idea supported by histological examination of AD brains [1,2,5] as well as metabolic imaging [14,36].

It appears that the initial reception and encoding of visual stimuli are relatively intact in AD. Rizzo et al. [41] did not find significant clinical dysfunction ascribable to retino-calcarine abnormalities in AD. Based upon event related potentials, Saito et al. [45] reported that in AD patients with mild dementia, early sensory processing appears to be intact, whereas impairment appears to be selective to high-level processing. Furthermore, visual acuity [20] and critical flicker fusion [9] in AD patients are comparable to age-matched control subjects. Among low-order visual functions that are impaired in AD are elevated contrast sensitivity thresholds [9,13,35,48], color deficits [10,24], stereoacuity deficits [9] and impaired motion perception [46]. High-order visual symptoms include impaired facial recognition [7,26,44], and impaired object recognition [6,22, 27,31,44].

Less is known about intermediate levels of visual processing in AD, including perceptual organization.

ISSN 0953-4180/03/$8.00 © 2003 – IOS Press. All rights reserved

Deficits have been found with visual grouping [25] as well as figure-ground separation [25,31]. Mielke et al. [34] found impaired performance in AD on the identification of fragmented pictures, which were associated with reduced metabolic activity in visual areas. Matsumoto et al. [29] found impairment in AD in the ability to integrate visual elements into global images. It has been suggested that impaired object recognition may stem from abnormal organization of the visual scene. Specifically, there appears to be improper use of discrete visual elements, either in segregating and identifying individual objects [7], or in simultaneously processing multiple elements to interpret the image [11]. Furthermore, it has been reported that AD may be accompanied by simultanagnosia, whereby patients are unable to integrate identifiable elements into coherent wholes [47].

Such perceptual impairments suggest a particular vulnerability in AD of integrating and organizing stimulus elements across the visual scene. It is therefore hypothesized that AD is accompanied by specific impairment of perceptual organization. Furthermore, because greater spatial separation of elements requires integration from more distal locations, impairment in perceptual organization should be greater with increased spatial scale. Because perceptual organization is subordinate to object recognition, it is further hypothesized that impairment in perceptual organization is associated with impairment in object recognition. Finally, based upon characteristics of other visual impairments in AD [33], as well as the regional variation in cortical neuropathology, it is hypothesized that impairment in perceptual organization will vary among subjects.

To test these predictions, perceptual organization based upon spatial relationships was compared between a group of AD patients and demographically matched elderly control subjects. Comparisons were also made between young and elderly healthy control subjects in order to assess the effects of aging on these capacities. Subjects received five tests of perceptual organization that were based upon different aspects of spatial relationships. Two of the tests consisted of identical displays that varied in spatial scale, in order to determine if greater relative distance among stimulus elements exacerbates impairment. Subjects also received a standardized test of facial recognition [4] in order to examine the relationship between perceptual organization and object recognition.

## 2. Methods

### 2.1. Subjects

Twelve patients diagnosed with probable AD, 20 elderly control subjects, and 20 young control subjects participated in the study. All participants reported to have no history of significant ophthalmologic disorder, and were verified to have a best corrected 14$''$ visual acuity of 20/25 or better (Snellen). The diagnosis of probable AD was based upon the diagnostic guidelines specified by the NIA [21] and the NINCDS-ADRDA [30]. The level of dementia for AD subjects was evaluated with the information, memory, and orientation section of the Blessed Dementia Scale (BDS), and ranged from 4 to 21 (mean = 12.7, maximum possible score = 37). AD patients ranged in age from 55 to 84 years (mean = 69.3). In compliance with regulations of the Institutional Review Board for Human Research, all subjects provided informed consent before participating in the study. Elderly control subjects consisted of community volunteers who ranged from 65 to 88 years (mean = 73.0). BDS for elderly controls ranged from 0 to 3 (mean = 1.7). AD patients did not differ significantly from elderly control subjects in age ($t(28) = -1.178$; $p > 0.1$) or years of education ($t(28) = -0.247$, $p > 0.1$). Young control subjects consisted of college students who participated in the study in order to fulfill course requirements.

### 2.2. Procedure

All participants received five psychophysical tests of perceptual organization, as well as the Benton Facial Recognition test. In order to assess visual capacities relatively uncontaminated by general dementia, testing procedures were designed to minimize demands on memory, language, and other high-order cognitive processes. Furthermore, all test conditions employed a forced-choice procedure, thereby eliminating possible response bias.

Subjects fixated a central target on a computer monitor at a viewing distance of 46 cm. For each trial, a stimulus appeared briefly on the monitor. Stimuli consisted of arrays of white squares on a dark background, covering a 19.3° square field. Stimuli were presented for a duration of 150 msec, thereby precluding the possibility of multiple fixations. Following stimulus presentation, subjects indicated verbally which of two possible patterns were formed by the stimulus. Responses were entered into the computer by the experimenter. Reaction time was not a factor, and subjects were instructed to maximize the accuracy, and not the speed, of their response. For each test, subjects first received a demonstration, and then a series of practice trials in order to become familiar with the stimulus and procedure. Following the demonstration and practice, threshold measurements were made. Stimulus generation, data collection, and contingency algorithms were controlled by computer.

Each of the five tests of perceptual organization (Proximity, Alignment, Glass Patterns, Large Shapes, and Small Shapes) were used to examine a different component of spatial relationships.

*Proximity.* Proximity is a strong cue for perceptual organization in which proximal elements tend to be perceptually grouped [42]. For the Proximity condition, stimuli consisted of a grid in which elements were more proximal along either the vertical or horizontal orientation (Fig. 1(a)). Elements along the more proximal orientation tended to be perceptually organized as a series of lines. For each trial, subjects indicated whether the stimulus appeared to be a series of vertical or horizontal lines.

*Alignment.* The test of Alignment employs the tendency for elements that form smooth lines to be perceptually grouped (Gestalt principle of Good Continuation) [39]. Stimuli consisted of a grid of elements. Elements along either the vertical or horizontal orientation were aligned, whereas elements along the alternate orientation were randomly offset (Fig. 1(b)). Elements along the aligned orientation tended to become perceptually grouped, forming a series of lines. Subjects indicated whether each stimulus appeared to be a series of vertical or horizontal lines.

*Glass Patterns.* Glass Patterns are geometric patterns generated by a systematic displacement of otherwise randomly distributed points. By superimposing the original and transposed points, the perception of a coherent pattern emerges from the random array [28]. For this condition, an array of randomly distributed elements was rotated about a single location. These stimuli elicited the perception of concentric, fragmented circles that contained a central area (Fig. 1(c)). For each trial, the central area of the array was displaced either to the left or to the right. Subjects indicated the position of the central area as either left or right.

*Large Shapes.* Stimuli consisted of an array of elements that formed the fragmented shape of either a square or a diamond (Fig. 1(d)). The size of the large shapes was 12° of visual angle. The fragmented shape was superimposed upon randomly distributed elements that served as visual noise. Subjects indicated whether the stimuli contained a square or a diamond.

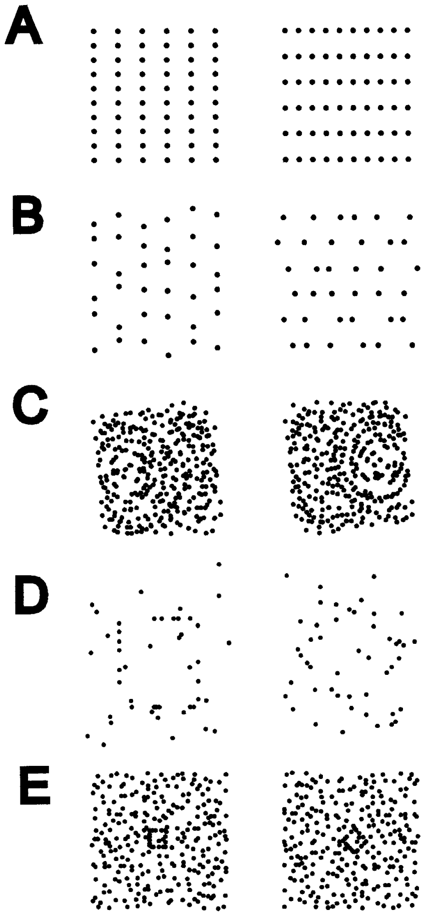

Fig. 1. Examples of stimulus types used in each of the five perceptual organization tests. For (A) Proximity Test and (B) Alignment Test, subjects discriminated between horizontally or vertically grouped dots. For (C) Glass Patterns Test, subjects indicated whether the central tendency was towards the left or right. For (D) Large Shapes Test and (E) Small Shapes Test, subjects indicated whether dots formed a square or a diamond.

*Small Shapes.* The Small Shape condition contained the same number of elements as the Large Shape condition, but differed in the mean separation among elements as well as the size of the shapes (Fig. 1(e)). For the Small Shapes condition, the square and diamond were 2.5° across a side. Subjects again indicated whether the stimuli contained a square or a diamond.

### 2.3. Threshold measurements

For each trial, one of the two possible stimulus patterns was randomly selected by computer, and subjects indicated the manner in which the stimulus appeared to be organized. Responses were made by means of a two-alternative forced-choice procedure.

Psychophysical thresholds were determined by means of a descending Method of Limits. Each series began with a strong cue for perceptual organization. Across trials, the stimulus cue upon which perceptual organization was based was progressively diminished until subjects selected the alternate (non-cured) grouping pattern. For Proximity, the relative difference in element separation was progressively reduced, thereby increasing the similarity between the vertical and horizontal orientation. For Alignment, the degree of misalignment was progressively reduced, thereby also making both orientations more similar. For Glass Patterns, the displacement between element pairs was made progressively more random, which obscured the position of the central area. For Large and Small Shapes, the amount of visual noise was progressively increased, thereby obscuring the perception of the embedded forms. Thresholds were based upon eight descending series. Thresholds represented the level at which the spatial relationship no longer served as a cue for grouping.

### 2.4. Control conditions

Each psychophysical measurement was accompanied by a control condition that monitored subjects' ability to discriminate figures constructed of solid lines. Performance on the control conditions provide an assessment of subjects' ability to understand the requirements of the tasks, to perceive and discriminate each pair of stimuli, and to respond appropriately. Impaired performance on experimental conditions is therefore attributable to perceptual deficits, and not other cognitive factors.

### 2.5. Benton Facial Recognition

Subjects viewed cards on which several photographs of faces were depicted. Subjects matched the face at the top of the card (standard) to one or more of 6 faces depicted below. Three sets of cards were used: the matched faces were front views, side views, and front views under different lighting conditions. Scores were based upon the number of correct matches out of 54 possibilities.

## 3. Results

For all measurements, raw scores were transformed to a common scale such that the highest possible score equaled 100% correct, and the lowest possible score equaled 50% (chance responding for a two-alternative forced-choice procedure). For example, for the Large and Small Shapes, the lowest possible score was 0, whereas the highest possible score was 18 (based upon the level of noise). A transformation of (raw score * 50/18) $+$ 50 converted a raw score of 18 to 100% and a raw score of 0 to 50% correct. For Benton Facial Recognition, the highest possible score was 27 (1 correct for each of the first 6 pages, and 3 correct for each of the last 7 pages), whereas chance performance was a score of 11.5 (choosing 1 of 6 possibilities in each of the first 6 pages $=$ probability of 0.67 for 6 selections; and choosing 3 of 6 possibilities for each of the last 7 pages $=$ probability of 0.50 for 21 selections).

Separate analyses compared performance between young and elderly control subjects (effects of aging), and between AD patients and elderly control subjects (effects of AD). In each case, group differences on the five tests of perceptual organization was assessed by means of a mixed-model multivariate analysis of variance (ANOVA). Bonferroni correction for multiple test chance effects was applied. Two AD patients (BDS $=$ 14 and 21, respectively) were unable to perform the control tasks and were therefore excluded from further testing. All other subjects performed at 100% accuracy for control tests. Subject group means for each test are presented in Fig. 2.

## 4. Effect of aging

The young and elderly control groups differed significantly (main effect of subject group: $F(1,38) = 38.82,\ p < 0.01$) as did performance across tests (main effect of test: $F(4,152) = 36.15,\ p < 0.01$). Furthermore, a significant interaction was found between subject group and test $(F(4,152) = 5.62,\ p < 0.01)$. To interpret the interactive effect, a Tukey HSD test was performed. For the tests of Alignment, Large Shapes, and Small Shapes, scores for the elderly control group were significantly lower than those for the young control group, whereas young and elderly control subjects did not differ significantly on the tests of Proximity and Glass Patterns (HSD $= 5.14,\ p > 0.05$).

Young and elderly control subjects did not differ significantly on the Benton Facial Recognition test

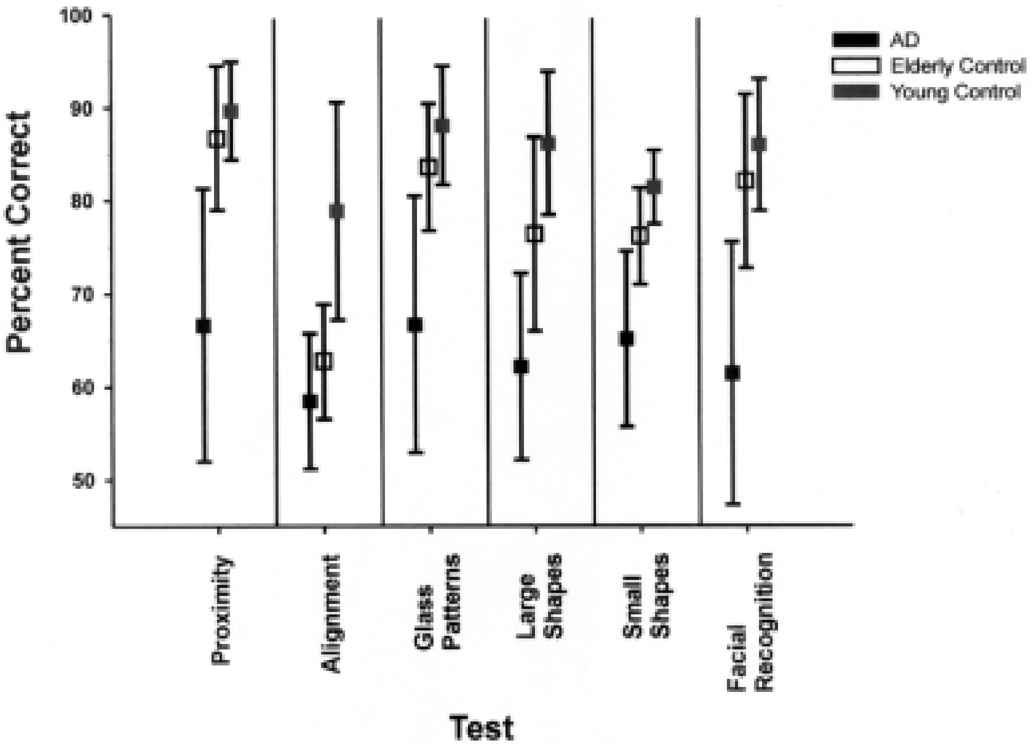

Fig. 2. Group performance on each of the five perceptual organization tests as well as Benton Facial Recognition. Symbols indicate group means, and error bars represent one standard deviation.

$(t(38) = -1.48,\ p > 0.1)$. For the young and elderly control subject groups, no single test of perceptual organization correlated significantly with performance on the Benton Facial Recognition test.

A discriminant analysis was performed to derive the weighted combination of the five perceptual organization tests that provided the greatest discrimination between subject groups. It was found that the relative importance for each test in discriminating subject group ranked from the test of Alignment (canonical coefficient = 0.831), to Large Shapes (coefficient = 0.438), to Small Shapes (coefficient = 0.283), to Glass Patterns (coefficient = 0.142), to Proximity (coefficient = $-0.039$). Overall, the discriminant analysis was highly accurate in predicting subject group based upon perceptual organization tests, placing 18 of 20 elderly control subjects, and 18 of 20 young control subjects in the correct group.

## 5. Effects of AD

With the exception of the Alignment test, in which performance declined significantly with age, AD patients were impaired in perceptual organization relative to elderly control subjects. A significant main effect was found between the AD and elderly control groups $(F(1, 28) = 31.93;\ p < 0.01)$ as well as among the five tests of perceptual organization $(F(4, 112) = 19.92;\ p < 0.01)$. A significant interactive effect of subject group by test was also found $(F(4, 112) = 4.59,\ p < 0.01)$. Follow-up analysis of the interaction indicated that AD patients were impaired on all five tests of perceptual organization except for the Alignment test (HSD = $6.2,\ p < 0.05$).

A separate ANOVA was performed on the tests of Large and Small Shapes. Contrary to the hypothesis, the degree of separation among stimulus elements did not exacerbate impairment in AD. AD patients were impaired on both conditions at comparable levels. A main effect of subject group was found $(F(1, 28) = 17.07,\ p < 0.01)$, whereas the main effect of test $(F(1, 28) = 0.92,\ p > 0.1)$ as well as the interaction between subject group and test $(F(1, 28) = 1.24;\ p > 0.1)$ were not significant.

AD and elderly control subjects differed significantly on the Benton Facial Recognition test $(t(28) = -4.80,\ p < 0.01)$. For the AD group, performance on

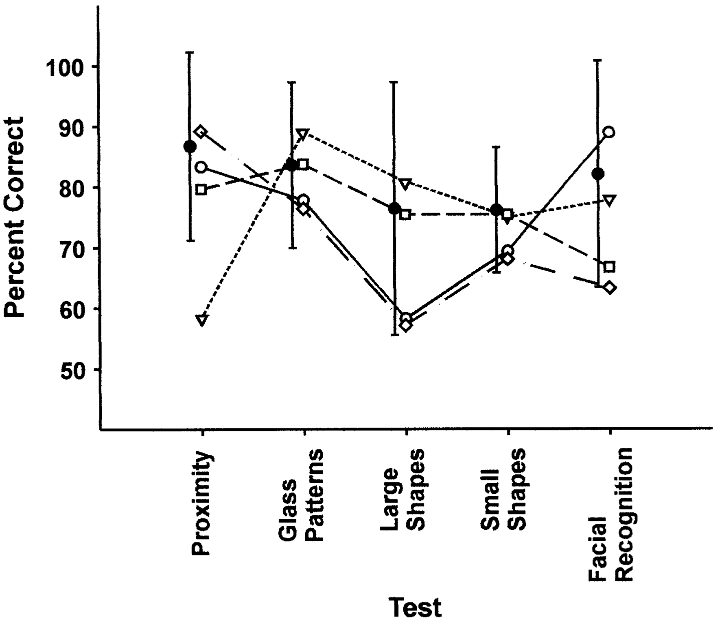

Fig. 3. Individual performance of four AD subjects who scored within two standard deviations of Elder Control subject's mean (with one exception on the Proximity test). Solid circles represent mean of Elderly Control subjects, and error bars represent two standard deviations.

the Benton Facial Recognition test correlated significantly with Glass Patterns ($r = 0.81,\ p < 0.05$).

For the AD group, no test of perceptual organization, nor the Benton Facial Recognition test, correlated significantly with the level of dementia. Discriminant analysis ranked the relative importance of each test for discriminating subject group from the test of Proximity (canonical coefficient = 0.609), to Large Shapes (coefficient = 0.337), to Glass Patterns (coefficient = 0.319), to Small Shapes (coefficient = 0.141), to Alignment (coefficient = $-0.117$). The discriminant analysis was highly accurate in predicting the subject group for elderly control subjects, placing 18 of the 20 subjects in the correct group. The discriminant function was less accurate in predicting group membership for AD patients, placing only 6 of the 10 subjects in the correct group.

For each of the tests, several of the AD patients performed at levels that were within the distribution of elderly control subjects, whereas others fell below this distribution. In order to determine whether this pattern reflected individual performance, scores for individual AD patients were tracked across tests. The test of Alignment was not used in this analysis because it was shown to be insensitive to AD. Four of the 10 AD patients fell within normal limits (within two standard deviations from the mean) of elderly control subjects on all perceptual organization tests (with one exception). Furthermore, all of these subjects performed normally on the test of facial recognition (Fig. 3). A second category of patients fell below normal limits on all tests, including facial recognition (Fig. 4). A third category of subjects had mixed performance, demonstrating impairment on at least two. Subjects from the mixed performance category were also impaired on facial recognition. These results demonstrate uniformity in performance across perceptual organization abilities for most AD patients. Furthermore, in most cases, performance on perceptual organization reflected that of facial recognition.

## 6. Discussion

These results indicate that perceptual organization based upon spatial relationships is impaired in AD, and this impairment is accompanied by deficits in facial recognition. These perceptual capacities also partially decline with age. Elderly control subjects were impaired on three of the five tests of perceptual organization, although no decline was observed for facial recognition. These results are not ascribable to cognitive impairment other than perceptual in that all participants performed accurately in control conditions, demonstrating that subjects understood the nature of the task, and were able to perform the perceptual discrim-

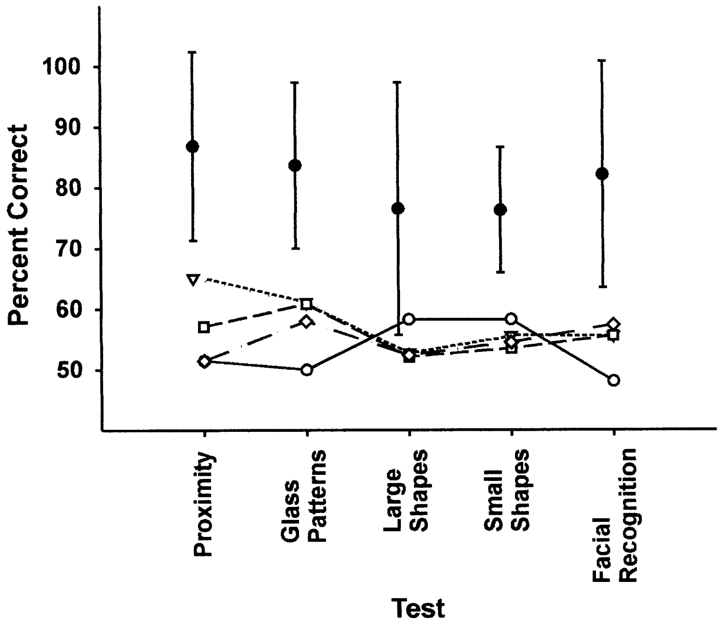

Fig. 4. Individual performance of four AD subjects who scored below two standard deviations of Elderly Control subject's group mean (one exception with borderline performance). Solid circles represent mean of Elderly Control subjects, and error bars represent two standard deviations.

inations employed in the psychophysical procedures. Furthermore, the forced-choice procedure ensured that selection bias did not impact results.

The level of functional decline differed across the five tests of perceptual organization. Furthermore, the pattern of decline found for aging differed from that found for AD. These results demonstrate variance in the mechanisms by which spatial relationships are used to establish perceptual organization. Proximity is a strong cue for perceptual organization in which processing occurs relatively rapidly (mean processing time of 87.6 ms) [23], suggesting a low-order, automatic process. Discriminant analysis indicated that perceptual organization by Proximity is least vulnerable in aging, although most vulnerable in AD. Alternatively, perceptual organization by Alignment requires significantly longer processing time (mean time of 118.8 ms) [23], suggesting a more computationally intensive mechanism. Performance on the Alignment test declined significantly with age. Furthermore, several young control subjects performed at levels comparable to elderly subjects (Fig. 2). These results suggest that the more elaborate processing necessary for the Alignment test is vulnerable to degradation by sources other than AD, whereas the more elemental processes used in the Proximity test serves as an effective predictor of AD.

Glass Patterns have been used to investigate global perceptual processing [38]. A central tendency becomes apparent by integrating relationships among local sets of stimulus elements. This type of global analysis did not decline with aging, but was affected by AD. Furthermore, performance on Glass Patterns was the only perceptual organization test to correlated with performance on facial recognition in AD. Perceptual organization of Glass Patterns, perhaps more that any of the other tests used here, is reliant upon global analysis across a broad region of the stimulus. Spatial relationships within local areas do not provide adequate indicators of the central region. This type of stimulus therefore appears to be well suited to identify perceptual organizational deficits in AD.

The tests of Small and Large Shapes consisted of identifiable figures embedded within visual noise. Similar types of stimuli have been used to examine perception of incomplete figures, in which deficits have also been reported for AD [40]. Processing such stimuli places heavy demands on perceptual organizational capacities, requiring proper assignment of stimulus elements to either the figure or noise. Improper assignment diminishes the capacity to identify the already impoverished figure.

A comparison between Small and Large Shapes tests was used to examined the effect of anatomical separation among cortical sites. Increased separation among stimulus elements corresponds to greater separation among activated neurons. Although deficits

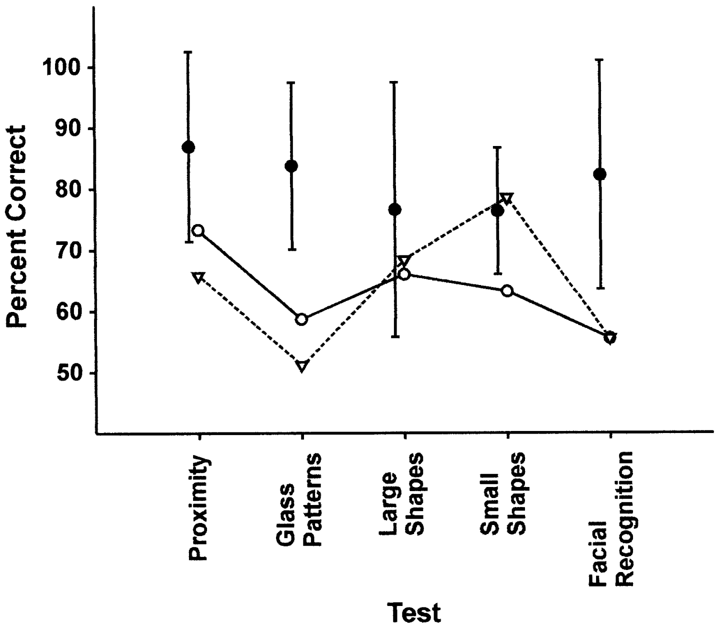

Fig. 5. Individual performance of two AD subjects who demonstrated mixed performance across tests. Solid circles represent mean of Elderly Control subjects, and error bars represent two standard deviations.

were found for both conditions, performance remained invariant across scalar differences. This effect may reflect changes in receptive field size as neural representations are transformed across cortical regions.

Perceptual organization occurs at an intermediate level of visual processing, organizing the visual scene in preparation for object recognition. Because this function is subordinate to object recognition, deficits at the level of perceptual organization should disrupt recognition ability, or exacerbate existing impairment. This effect was not observed for aging. Performance on the Benton Facial Recognition test did not differ significantly between young and elderly control subjects, nor did any test of perceptual organization correlate to performance on the facial recognition test for the elderly control group. AD subjects, however, performed significantly poorer on Benton Facial Recognition than elderly control subjects. Although performance on facial recognition by AD subjects correlated only with tests of Alignment and Glass Patterns, subgroup analysis indicated that those AD subjects who were impaired on facial recognition were also impaired on tests of perceptual organization. Although the relationship between perceptual organization and object recognition in AD requires further investigation, these results demonstrate a link between these two levels of visual processing.

Visual impairment was not evident in all AD subjects, but occurred in a subgroup of patients. In this regard, discriminant analysis based upon tests of perceptual organization was inaccurate in predicting subject group membership. Furthermore, the incidence and degree of visual impairment did not correlate with level of dementia as measured by BDS, which is weighted heavily by cognitive and memory items, but instead appeared to be idiosyncratic to individual patients. This pattern of results is characteristic of other visual capacities in AD [33]. Variation in visual impairment across AD patients suggests differential patterns of cortical pathology, specifically, whether or not neuropathological changes have invaded visual areas. Consistent with this prediction are reports in which patients who demonstrated clinical symptoms of either visuospatial or recognition impairment were later identified as having significant neuropathology in posterior parietal or inferotemporal cortex, respectively [15,17].

Perceptual organization requires the integration of information. Long corticocortical projections appear to be particularly vulnerable in AD. Rogers and Morrison [43] examined neuropathological changes in clinically and neuropathologically confirmed AD patients. The regional and laminar plaque distribution found in these studies indicated that neuropathological changes in AD most greatly affect cross-cortical connections [16]. The authors noted that the specific loss of this subset of neurons would result in reduced effectiveness of distributed processing within the cortex, which is likely reflected by reduced abilities on complex cognitive functions.

In the tests of perceptual organization used here, the configuration of the stimulus was defined by means of element position, referred to as Type P configuration [37]. Many other stimulus features, such as color, size, luminance, and motion coherence, also serve as cues for perceptual organization. For each of these stimulus features, disruption at the level of either basic processing or that of perceptual organization will impact an observer's ability to identify objects and distinguish them from their backgrounds. Analysis of processing constituent feature in this manner is beneficial in understanding the characteristics of visual impairment in AD. Identifying specific characteristics of visual impairment can aid in designing effective visual environments for the care of AD patients. Furthermore, understanding the constituent visual symptoms in AD will facilitate investigation of the impact of visual impairment on other cognitive functions, such as memory.

## Acknowledgements

This study was supported by NIH grant R15 AG13758.
